# Breast Milk Expression Frequency and Production, Na Concentrations and Na:K Ratios in the First 4 Weeks After Preterm Birth

**DOI:** 10.3390/nu18091418

**Published:** 2026-04-30

**Authors:** Sharon Lisa Perrella, Emma-Lee Anderton-May, Xuehua Jin, Donna Tracy Geddes

**Affiliations:** 1 School of Molecular Sciences, The University of Western Australia, Crawley, WA 6009, Australia; xuehua.jin@uwa.edu.au (X.J.); donna.geddes@uwa.edu.au (D.T.G.); 2 UWA Centre for Human Lactation Research and Translation, Crawley, WA 6009, Australia; 3 ABREAST Network, Perth, WA 6000, Australia; 4 Neonatology Clinical Care Unit, King Edward Memorial Hospital, Subiaco, WA 6008, Australia; emma-lee.anderton@health.wa.gov.au

**Keywords:** lactation, breast milk, preterm, premature birth, milk production, breast milk expression, secretory activation, biomarkers, low milk supply

## Abstract

**Background/Objectives**: Low milk production is more prevalent after preterm birth and may be associated with infrequent milk expression, delayed secretory activation and elevated milk biomarkers including sodium (Na) and sodium–potassium ratio (Na:K). This study aimed to describe milk production, expression frequency, and milk biomarkers (Na and Na:K) in the first 4 weeks and explore associations in the first 2 weeks after preterm birth. **Methods**: Women who birthed at 28–34 weeks of gestation provided milk expression data and milk samples every second day from birth to Day 10, then every third day until infant transfer/discharge from the neonatal unit. Lactation characteristics and milk Na and Na:K across the first 4 weeks were described, and associations between milk production, expression frequency and milk biomarkers were examined. **Results**: In a sample of N = 44 women that maintained a median expression frequency of 6–7 × 24 h, temporal patterns in milk Na and Na:K were similar to those observed after birth at term, and a milk production ≥ 600 mL/24 h was achieved by 61.5% on Day 13. One third of women experienced delayed secretory activation. Expression volumes on Day 4 were associated with milk production on Day 13 and Day 16 (both *p* < 0.001). **Conclusions**: Our findings suggest that low expression volumes in the days after preterm birth may indicate women at risk of low milk production. Further research is needed to determine the predictive value of early expression frequency and milk composition on subsequent milk production.

## 1. Introduction

Human milk is critical in the health of preterm infants as it prevents or reduces the severity of many health complications. Specifically, feeding of mother’s own milk (MOM) during the neonatal nursery admission provides immunologic benefits and is associated with significant risk reductions for retinopathy of prematurity and necrotising enterocolitis [1,2,3]. A dose–response effect is noted, with higher proportions of MOM feeding associated with a lower risk of necrotising enterocolitis, white matter injury and dysmaturation, and enhanced neurodevelopmental outcomes [3,4].

While optimisation of maternal milk production and continued breastfeeding are important strategies for preterm infant health, low milk production is more prevalent after preterm birth [5,6]. This limits the availability of MOM and is associated with a shorter breastfeeding duration [7,8]. Published thresholds for adequate milk production range from ≥500 mL/24 h by Day 14 postpartum to ≥750 mL/24 h by Day 10 [9,10,11]. Milk production ≥ 500 mL/24 h on Day 14 is associated with full MOM feeding of preterm infants at discharge; however, their milk volume requirement is higher and similar to term-born infants beyond term-corrected age [9,12]. As milk production is typically 600–625 mL/24 h by Day 7 and ~788 mL/24 h by 4 weeks after birth at term, a higher threshold for low milk production is warranted [13,14].

Early expression frequency and milk production are positively associated with later milk production [11,15,16]. An expression frequency of 8/24 h is recommended as longer expression intervals are associated with a lower milk synthesis rate with consequent lower milk production and delayed secretory activation (SA) [17,18,19]. Maternal health factors including gestational hypertensive disorders, gestational diabetes mellitus (GDM) and preterm birth have also been associated with delayed SA and reduced milk production [20,21,22,23].

Secretory activation is preceded by changes in milk biomarker concentrations such as rapid reductions in sodium (Na) and protein at 32–40 h postpartum, with SA onset characterised by maternal perceptions of breast fullness, copious milk production and milk Na < 16 mmol/L at 60–70 h [24,25]. Delayed SA beyond 72 h is associated with subsequent low milk production and breastfeeding cessation prior to discharge from the neonatal unit [26,27,28]. Criteria for identifying SA vary between settings and maternal reporting of its timing is typically imprecise [25]. In pump-dependent mothers, it is considered to have occurred when two consecutive pumping sessions yield ≥ 20 mL [29]. More recently, it has been shown that milk biomarkers may provide a useful indicator of SA and subsequent milk production [29,30].

Timely secretory activation is critical in the establishment of milk production and is characterised by reducing milk chloride and Na and sodium-to-potassium ratio (Na:K) [31]. Delayed SA is reflected in elevated milk Na > 12–16 mmol/L and Na:K > 1.0 beyond Day 4 and is associated with subsequent low milk production [30,32]. Following preterm birth, elevated milk Na and Na:K on Day 3 are associated with failure to achieve a milk production of ≥500 mL/24 h by Day 14 [6]. While Na:K has been used in research, point of care (POC) milk Na alone may be adequate and more clinically feasible for the early identification of mothers at risk of delayed SA and compromised milk production [6,25,30].

Milk expression frequency, perinatal complications and the timing of SA may all impact the availability of MOM for hospitalised preterm infants. Examination of expression frequency, milk yield and Na in the weeks after preterm birth can aid in identifying women requiring additional lactation support.

The primary aim of the study, reported elsewhere, was to determine whether elevated milk Na:K and Na are associated with mastitis in mothers of preterm infants [33]. In this paper we report the secondary aims of the study, i.e., to describe SA, 24 h milk production, expression frequency and milk Na:K and Na in the first four weeks after preterm birth. Associations between milk Na and Na:K with expression frequency and milk production are also explored.

## 2. Materials and Methods

An observational study of milk Na and K and Na:K, onset of SA and milk production was conducted in mothers of preterm infants born ≤34 weeks gestation. Women provided background and lactation history data, and serial milk sampling and recording of 24 h expression volumes were completed throughout the infant’s neonatal unit admission.

### 2.1. Participants

The study was conducted in the neonatal unit of King Edward Memorial Hospital, Perth, Western Australia. It is a tertiary neonatal centre with 30 intensive care beds and 50 special care beds. In this study setting, all mothers of infants admitted to the neonatal unit were advised to hand-express within 2 h of birth and perform simultaneous pumping 7–8 × 24 h. They were loaned a hospital-grade electric breast pump and kit for the duration of their infant’s admission. Mothers that gave birth at 29–34 completed weeks of gestation who were expressing milk for their infant/s were provided with verbal and printed study information and invited to participate in the study within 48 h of birth. We included mothers ≥18 years of age who intended to visit their infant daily. Exclusion criteria included mothers that were non-English speaking, required hospitalisation beyond routine postpartum care or had complex health and/or psychosocial issues that prevented regular breast expression and/or daily visits to the neonatal unit. Researchers confirmed eligibility and obtained written informed consent prior to participation. This study was approved by the Women and Newborn Health Service Human Research Ethics Committee (#: RGS000103) and The University of Western Australia Human Research Ethics Committee (#: RA/4/1/9308). Informed written consent was obtained from all participants.

Sample size determination was based on the primary aim of the study, i.e., to determine whether elevated milk Na or Na:K was associated with mastitis in mothers of hospitalised preterm infants, as previously reported [33]. It was estimated that 62 participants were required to ensure a 95% chance of seeing at least two cases of elevated Na:K, as calculated using a simulation model with *p* = 0.05 to a power of 95%.

### 2.2. Data Collection

Demographic data were collected on the day of recruitment. Participants used a diary that is routinely provided in the study setting to record all breast expression volumes and breastfeeding episodes for each day of the neonatal unit admission. Serial milk sampling for Na and K analysis was performed every second day from Days 2 to 10, then every third day until infant discharge or transfer from the study hospital. Secretory activation was considered as milk Na:K < 1.0 or expression volume ≥ 20 mL × 2 in 24 h per breast [26,29,34]. Delayed SA was indicated if these criteria were not met by Day 4. For this study, low milk production was considered as <600 mL/24 h at or beyond Day 13. The daily milk expression volume was considered as the participant’s 24 h milk production volume as breastfeeding experiences were infrequent in the first 4 weeks. Further, it is known that milk transfer volumes are typically <5 mL in infants < 36 weeks corrected gestational age [35]. Participants were contacted at 4 weeks and 8 weeks postpartum and asked whether their infant was receiving only MOM (full MOM feeding), MOM and commercial milk formula (mixed fed), or only commercial milk formula.

Bilateral milk samples of minimum 0.3 mL were collected by the participant or the researcher according to the participant’s preference using aseptic techniques. Milk Na and K concentrations were measured in duplicate using handheld ion selective metres (Horiba©, Kyoto, Japan). Results were averaged and used to calculate milk Na:K. The ion metres have been validated for use in human milk [36,37,38]. Milk sample collection was omitted if the participant was unable to express the required volume, unable to meet her infant’s milk volume requirements and/or expressed concern about providing samples. When unable to meet with the researcher, the participant performed sample collection as per the study protocol and samples were stored in a research freezer at −20 °C for later analysis [39].

### 2.3. Statistical Analysis

Descriptive analyses were performed for all participants that provided any milk samples up to Day 28. For a subgroup of participants that had complete data available on Days 4 and 13 or 16, analysis was performed to determine associations between milk Na, Na:K, expression frequency and milk production on Days 13 and 16. Analyses were conducted using all available data at each time point without imputation.

Medians (25th, 75th percentiles) and frequencies (percentages) were used for descriptive statistics. Univariable linear regression analyses were performed to examine associations between milk production, expression frequency, Na, and Na:K. Univariable logistic regression analyses were used to assess associations with binary outcomes, such as total milk production < 600 mL or milk production per breast < 300 mL. In addition, linear mixed-effects models were used to examine longitudinal associations between milk production per breast and Na or Na:K over time, including postnatal day as a fixed effect. A random intercept for participant was included to account for within-participant correlation arising from paired breast-level observations. Given the exploratory nature of the secondary analyses, associations between predictors and outcomes were assessed using univariable models to evaluate their individual clinical relevance. Multivariable modelling was not undertaken due to the limited sample size and risk of overfitting. As there is emerging research on potential roles of maternal health factors in delayed SA and low milk production, we subsequently looked at GDM, PE, and BMI. The significance level was set at *p* < 0.05, and all analyses were carried out in R Statistical Software 4.2.2 (R Foundation for Statistical Computing, Vienna, Austria).

## 3. Results

**Figure 1:**
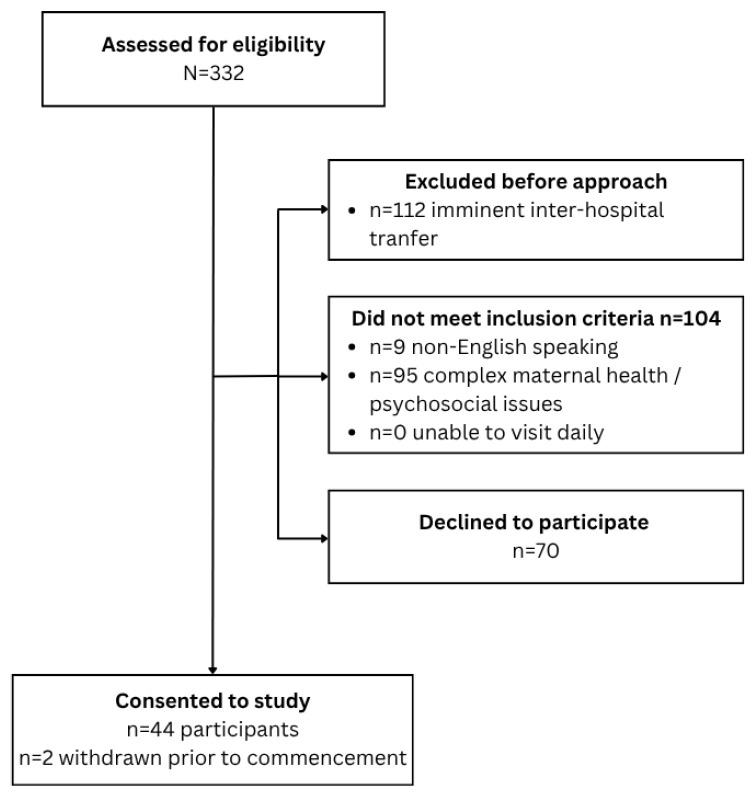
Recruitment flow diagram.

Participants were recruited between October 2017 and April 2019. This study aimed to recruit 65 participants. Of 332 mothers screened for study eligibility, 46 consented to participate, of whom 2 withdrew due to transfer to another hospital prior to commencement of data collection (Figure 1). Recruitment ceased prior to achievement of the required sample size (N = 65) due to an unexpectedly low recruitment rate and timeframe limitations. Data and samples were obtained for 44 participants and their 48 infants (39 singletons, 5 sets of twins). Three participants withdrew from the study due to other commitments but agreed to their data being included in the analysis.

A total of 660 milk samples (330 paired samples from 44 participants) were collected for milk biomarker analysis. Samples collected during episodes of mastitis beyond Day 8 (n = 4 episodes, 6 samples) were excluded as these are associated with elevated Na and Na:K and are reported elsewhere [31]. One pair of samples was excluded due to aberrant K readings, and all samples from one participant were excluded due to inconsistent timing of collection. Across Days 2–28, the number of analysed samples per participant ranged from 1 to 22, with a median of 13 (IQR: 7–18). The number of samples per day varied, with the greatest sampling density on Days 4, 6, and 8 and fewer samples at later time points (Table A1). Approximately 10% of samples were stored at −20 °C prior to initial analysis.

### 3.1. Participant Characteristics

Participants typically identified as being of Australian ancestry (84%), with the remainder identifying as British (11%) or Asian (5%). The cohort included 20 (46%) multiparous women of which 18 had previously breastfed. Reported health factors that have been associated with low milk production included breast augmentation surgery (n = 1, 2%), no breast growth in pregnancy (16/37, 43%), and cigarette smoking (5/41, 12%). Participant characteristics are reported in Table 1.

### 3.2. Expression Frequency

Milk expression was typically initiated within 6 h of birth (n = 28, 64%) of which 16 (36%) commenced within 1 h. A further 12 (27%) commenced > 6–24 h and 4 (9%) commenced after 24 h. The median expression frequency was 6–7/24 h across the first 28 days (Table A2).

### 3.3. Secretory Activation and 24 h Milk Production Volumes

Day 4 milk production data for each breast (‘per breast’) were available for n = 38 participants, with milk Na and Na:K available for n = 37 from 73 breasts. Delayed SA was identified in ~30% of participants, regardless of whether it was considered as a low expression volume, or elevated Na or elevated Na:K (Table 2). This prevalence was not different for participants with GDM (n = 4) or for those with gestational hypertensive disorders (n = 11). Discrepancies between breasts were sometimes noted. One participant reported Day 4 bilateral elevated milk Na and Na:K but 24 h expression volumes of 35 mL and 175 mL per breast. Of the ten participants with low Day 4 expression volumes and available milk biomarker data, five had unilateral elevated Na:K, one had unilateral elevated Na, and one had bilateral normal Na and Na:K.

**Figure 2:**
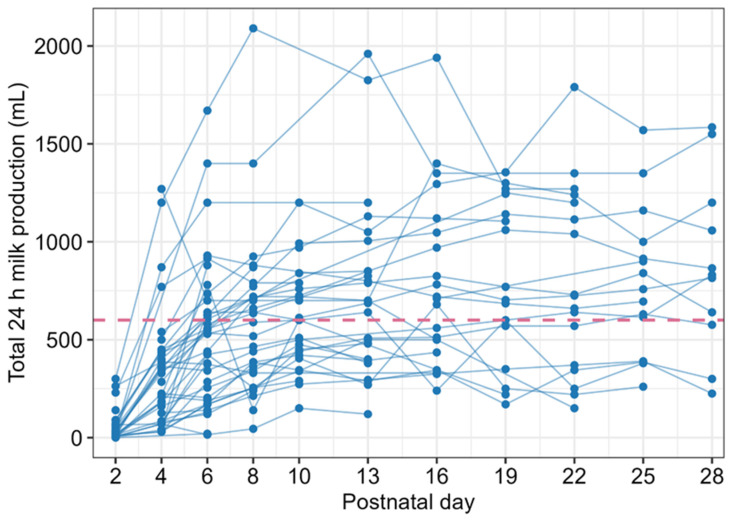
Total 24 h milk production in the first 4 weeks after preterm birth. Red dashed line indicates the 600 mL/24 h threshold for low milk production.

Median (Q1, Q3) total 24 h milk production increased from 22 (11, 65) mL on Day 2 to 532 (217, 685) mL on Day 6 and remained >600 mL from Day 10 onwards (see Table A2). By Day 13, 16 (61.5%) achieved a milk production ≥ 600 mL (Figure 2).

### 3.4. Milk Na and K Concentrations and Na:K Ratios

**Figure 3:**
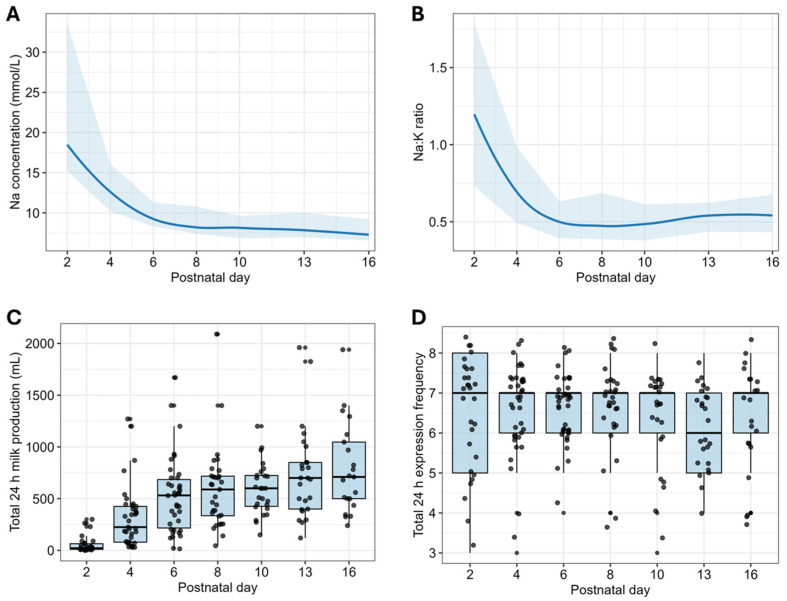
Milk Na concentration and Na:K ratio, 24 h milk production volumes and expression frequency in the first 16 days postpartum. (**A**) Milk Na depicted as median (25th, 75th percentiles) on Days 2 to 16. (**B**) Milk Na:K depicted as median (25th, 75th percentiles) for Days 2 to 16. (**C**) Total 24 h milk production volumes (mL) represented as box plots showing minimum, maximum and interquartile range for Days 2 to 16. (**D**) Total 24 h milk expression frequency represented as box plots showing minimum, maximum and interquartile range for Days 2 to 16.

Milk Na and Na:K rapidly declined between Days 2 and 6, with a gradual decrease thereafter (Figure 3A,B). The milk Na was 12.6 (10.2, 16.1) mmol/L on Day 4 and 8.4 (7.4, 10.8) mmol/L on Day 8, reducing further to 7.3 (6.6, 9.2) mmol/L by Day 16 (Table A2). The median milk K was ~20 mmol/L in the first week and reduced to 13.9 (12.3, 16.2) mmol/L by Day 16 (Table A2). Milk Na:K reduced from 1.2 (0.7, 1.8) on Day 2 to 0.7 (0.5, 1.0) on Day 4.

### 3.5. Associations Between Milk Na, Na:K, Expression Frequency and 24 h Milk Production Volume

With regard to milk Na and Na:K, no significant associations were observed between early Na or Na:K (Day 2 or Day 4) and milk production per breast at Day 13 or Day 16. However, both Na (β = −7.78; 95% CI: −10.04, −5.52; *p* < 0.001) and Na:K (β = −68.33; 95% CI: −92.81, −43.85; *p* < 0.001) showed consistent inverse associations with milk production per breast across the postnatal days, without time-dependent effects (Table 3).

Neither time to first expression nor expression frequency on Day 2 was associated with total milk production on Day 13 and Day 16. Higher expression frequency on Day 2 was associated with an increased likelihood of full MOM feeding at week 4 postpartum (OR = 1.94; 95% CI: 1.12, 3.37; *p* = 0.018).

With regard to total 24 h milk production, Day 2 production was positively associated with Day 13 (β = 3.81; 95% CI: 0.54, 7.07; *p* = 0.025) but not with Day 16 production (β = 1.54; 95% CI: −0.99, 4.07; *p* = 0.210). Similarly, Day 4 production was positively associated with production on both Day 13 (β = 0.80; 95% CI: 0.46, 1.14; *p* < 0.001) and Day 16 (β = 0.92; 95% CI: 0.52, 1.33; *p* < 0.001). Half of the participants (19/37, 51.4%) had a Day 4 production < 250 mL/24 h with subsequent low milk production (Figure 2), so further analysis was undertaken. A lower Day 4 production was associated with low milk production on Day 13 (OR = 0.98; 95% CI: 0.97, 1.00; *p* = 0.012) and Day 16 (OR = 0.99; 95% CI: 0.99, 1.00; *p* = 0.046).

### 3.6. Maternal Factors

Maternal BMI was inversely associated with milk production on Day 4 (β = −19.56; 95% CI: −38.06, −1.05; *p* = 0.039), Day 13 (β = −41.30; 95% CI: −77.46, −5.13; *p* = 0.028), and Day 16 (β = −56.89; 95% CI: −96.52, −17.25; *p* = 0.009). Higher BMI was also associated with an increased likelihood of low milk production on Day 16 (OR = 1.66; 95% CI: 1.01, 2.73; *p* = 0.047) and was inversely associated with the likelihood of full MOM feeding at week 4 (OR = 0.80; 95% CI: 0.69, 0.92; *p* = 0.002) and week 8 (OR = 0.85; 95% CI: 0.74, 0.97; *p* = 0.016) postpartum. No associations were seen between GDM (n = 4) or gestational hypertensive disorders (n = 11) and total milk production on Day 13 or Day 16.

## 4. Discussion

In a cohort of English-speaking mothers that gave birth at 28–33 weeks gestation and were able to visit their infants daily, SA was delayed in one third, and 16 (61.5%) achieved a milk production ≥ 600 mL by Day 13. The pattern of reduction in milk Na and Na:K was similar to that measured after birth at term [40], rapidly reducing in the first week then stabilising beyond Day 8 (Figure 3A,B). Milk production on Days 2 and 4, but not milk Na and Na:K, was positively associated with milk production on Days 13 and 16. Our findings demonstrate higher milk production and a more rapid completion of SA than those typically reported in studies on lactation after preterm birth. Further, the results highlight that for pump-dependent mothers after very preterm birth, greater emphasis should be placed on the first four days postpartum, with continued intensity for the first two weeks, to successfully establish lactation. Targeted support and monitoring of expression frequency and milk production volumes, together with serial milk Na assessments, may be useful in tracking SA and optimising outcomes for those at risk of low milk supply.

Initiation of expression ≤ 6 h of birth was achieved by approximately two thirds of participants and a median expression frequency was mostly maintained at 7/24 h across the first four weeks (Table A2). The timing of initiation was not associated with subsequent milk production. The prevalence of early initiation in this cohort contrasts with several published reports of ≤50% initiation ≤ 6 h of preterm birth [16,41,42,43,44]. Also, the observed median expression frequency was higher than a reported prevalence of expression frequency ≥ 7/24 h of 28.2% on Day 4, and <45% on Days 14 and 21 [44], and other reported median frequencies of ≤5/24 h [5,45,46]. Initiation of expression within 6 h of birth and an expression frequency of ≥7–8/24 h are recommended for pump-dependent mothers to facilitate SA and subsequent higher milk production volumes [19,43,45]. Frequent expression promotes SA as nipple stimulation elicits a transient surge in prolactin concentration to accelerate closure of the mammary epithelial cell tight junctions [47,48]. The prolactin response to breastfeeding is similar to that of simultaneous breast expression with an electric breast pump but significantly lower for hand expression, manual and battery-operated breast pumps [49]. Maintenance of frequent expression can be challenging after preterm birth, particularly when dealing with health complications, other family responsibilities and separation from the infant [50]. Therefore, it is imperative that after preterm birth, mothers receive support and instruction in milk expression with easy access to electric breast pumps, simultaneous pumping and optimised pumping patterns prior to SA [11,34,51,52].

More than two thirds of participants achieved SA by Day 4, with only slight variation according to the criteria used (Table 2). Our finding is similar to that of a recent systematic review reporting an overall 30% prevalence of delayed SA, as well as that reported after birth at 29–35 weeks gestation [20,32]. Several other studies have reported that preterm birth is associated with >50% prevalence of delayed SA [27,41,45,53]. This may be partly explained by differences in expression frequencies and methods, as well as in the prevalence of factors associated with delayed SA, such as elevated maternal BMI, GDM, gestational hypertensive disorders and unplanned Caesarean birth [20].

Milk Na and Na:K were inversely related to milk production on the day of sampling but not associated with subsequent milk production. Our findings contribute further evidence that milk biomarkers can be used to evaluate the onset of SA and responses to lactation interventions, while Na or Na:K alone is less useful in identifying those at risk of low milk production [25].

Expression frequency on Day 2 was not associated with milk production at Day 13 or Day 16 but was associated with an increased likelihood of full MOM feeding at week 4. The lack of association with milk production at 2 weeks contrasts with previous studies, with expression frequency ≤ 5/24 h associated with subsequent low production [18,19,45,54,55]. Our study was likely not adequately powered for analysis of this secondary study, and most participants expressed ≥5/24 h across the first month.

Early postpartum expression volumes were positively associated with subsequent expression volumes, whereby lower volumes on Days 2 and 4 were associated with low milk production at 2 weeks postpartum. Our findings are consistent with a study where delayed SA (defined as three consecutive expressions of ≥20 mL by Day 4) was associated with a lower milk production on Day 7 (median 160 mL vs. 300 mL, *p* < 0.001) and at the time of infant discharge [27]. Similarly, Parker et al. reported that earlier attainment of two consecutive expressions of 20 mL was associated with higher expression volumes across the first four weeks but not with milk Na or lactose concentrations [26]. Expression volumes in the days after birth are easily measured and may provide a useful indicator of risk for low milk production, signalling a need for targeted lactation support.

Given the evolving evidence for BMI as a risk factor for suboptimal lactation, we explored associations between maternal BMI and SA, milk production at 2 weeks and subsequent infant feeding outcomes [56,57]. BMI was inversely related to milk production on Days 4, 13 and 16, with an increased likelihood of low milk production on Day 16; infants were less likely to be fully MOM-fed at 4 and 8 weeks after birth. Larger prospective studies are needed to examine biochemical, psychosocial and lactation management factors that impact milk production in mothers with increased adiposity.

While many studies use a milk production goal of ≥500 mL/24 h at 2 weeks, this study’s threshold of ≥600 mL/24 h at 2 weeks was based on the fact that most very preterm infants are discharged home before 40 weeks of postmenstrual age and progress to the term-born infant’s milk volume requirement of >750 mL/24 h in the weeks after discharge [18,58]. As recent research suggests 708 mL/24 h as a threshold for low milk production, a 2-week milk production goal of ~700 mL/24 h would more likely support breastfeeding beyond the neonatal unit stay [59,60]. It is important to acknowledge that a full milk production ≥ 700 mL/24 h may not be achievable for some mothers as non-modifiable lactation risk factors are typically over-represented in the neonatal unit setting. Therefore, lactation risk screening should be completed to inform individualised counselling to optimise expression practices and subsequent milk production within the confines of any personal situations or conditions [21].

Limitations of this study include the exclusion of non-English speaking mothers and a high proportion of participants identifying as being of Australian ancestry. Given that Australia is a multicultural society, our findings may not be generalisable to the wider population. A further limitation of this study is the relatively low number of participants with impaired lactation in relation to other published studies. This likely limited the ability to detect statistically significant associations between elevated milk Na or Na:K and low milk production. Due to the exploratory nature of this secondary aim and the limited sample size, multivariable adjustment for potential confounders was not performed. Hence, these results should be interpreted as preliminary, and future studies with larger cohorts are required to confirm these associations using multivariable modelling.

## 5. Conclusions

Our findings suggest that monitoring of expressed milk volumes in the days after preterm birth can be used to identify mothers at risk of low milk production, while early postpartum milk Na and Na:K may be useful in tracking SA. Larger studies with a range of expression volumes and frequencies, lactation risk factors and milk composition are needed to further explore factors that may predict low milk production after preterm birth.

## Figures and Tables

**Table 1:** Participant characteristics.

Characteristic	n	Median (25th, 75th Percentiles) or n (%)
Maternal age (years)	44	30.5 (27.8, 34.0)
Body mass index (BMI)	33	26.2 (23.8, 34.1)
BMI ≥ 30.0		10 (30%)
Education—tertiary	42	17 (41%)
Intended BF ^1^ duration (months)	43	12 (12, 12) n = 31
“As long as I can/baby wants to”		12 (27%)
Pregnancy complications	44	
Gestational diabetes mellitus		5 (11%)
Pre-eclampsia		12 (27%)
PPROM ^2^		8 (18%)
Birth mode—Caesarean	44	25 (52%)
Birth gestation	44	31.6 (30.1, 33.0)
Female infant	44	22 (50%)

^1^ BF = breastfeeding; ^2^ PPROM = preterm premature rupture of membranes.

**Table 2:** Delayed secretory activation (SA) on Day 4 and associated 24 h milk production outcomes on Days 13 and 16 postpartum.

Delayed SA Criteria	n (%)	Day 13 Production (mL/24 h)	Day 16 Production (mL/24 h)
Milk expression per breast < 40 mL/24 h ^1^	10/37 (27.0%)	>600 1/10; 640 mL	>600 1/10; 680 mL
		≤600 5/10	≤600 3/10
		Missing 4/10	Missing 6/10
Milk Na ≥ 16 mmol/L	12/35 (34.4%)	>600 4/12	>600 3/12
		≤600 3/12	≤600 1/12
		Missing 5/12	Missing 8/12
Milk Na:K ≥ 1.0	11/35 (31.4%)	>600 3/11	>600 2/11
		≤600 3/11	≤600 2/11
		Missing 5/11	Missing 7/11

^1^ As the minimum expression frequency was 3/24 h, it follows that a 24 h milk expression volume < 40 mL per breast would result from two consecutive expressions < 20 mL.

**Table 3:** Associations between Na concentration/Na:K ratio and milk production per breast over time.

	Variable	Estimate (β)	95% CI	*p*-Value
Model 1	Na (mmol/L)	−7.78	−10.04, −5.52	<0.001
	Postnatal day	5.39	2.25, 8.54	<0.001
	Na × day	0.29	−0.06, 0.64	0.103
Model 2	Na:K ratio	−68.33	−92.81, −43.85	<0.001
	Postnatal day	7.61	4.55, 10.69	<0.001
	Na:K × day	2.54	−2.29, 7.35	0.304

## Data Availability

Given the sensitive nature of the data, and in line with the consent agreed with participants, restrictions apply to the availability of some or all data generated or analysed during this study. The corresponding author will, on request, detail the restrictions and any conditions under which access to some data may be provided.

## Abbreviations

The following abbreviations are used in this manuscript:

GDM	Gestational diabetes mellitus
Na	Sodium
Na:K	Sodium-to-potassium ratio
SA	Secretory activation

## Appendix A

**Table A1:** Number of participants who provided samples and milk Na and K concentrations and Na:K in the first 28 days after birth at 28–34 weeks gestation. Values are reported as median (25th, 75th percentiles).

Postpartum Time	n	Milk Na mmol/L	Milk K mmol/L	Milk Na:K
Day 2	19	18.5 (15.2, 33.5)	17.9 (13.4, 21.1)	1.2 (0.7, 1.8)
Day 4	35	12.6 (10.2, 16.1)	19.6 (16.2, 22.6)	0.7 (0.5, 1.0)
Day 6	33	9.2 (8.3, 11.3)	18.7 (16, 22.3)	0.5 (0.4, 0.6)
Day 8	32	8.4 (7.4, 10.8)	17.3 (15.3, 21.1)	0.5 (0.4, 0.7)
Day 10	26	8.3 (6.9, 9.6)	16.4 (14.3, 18.8)	0.5 (0.4, 0.6)
Day 13	27	7.8 (7, 10)	15.3 (13.3, 17.1)	0.6 (0.4, 0.6)
Day 16	25	7.3 (6.6, 9.2)	13.9 (12.3, 16.2)	0.5 (0.4, 0.7)
Day 19	19	6.5 (5.7, 7.4)	13.8 (12.3, 16.4)	0.5 (0.4, 0.5)
Day 22	18	6.3 (5.2, 8.3)	13.8 (12.9, 15.3)	0.4 (0.4, 0.5)
Day 25	16	6.9 (5.7, 8.5)	14.5 (11.8, 17.3)	0.5 (0.4, 0.6)
Day 28	11	6.1 (5.3, 7.7)	13.2 (11.5, 16.4)	0.5 (0.4, 0.6)

**Table A2:** Milk expression frequency and 24 h milk production volume in the first 28 days after birth at 28–34 weeks gestation. Values are reported as median (25th, 75th percentiles).

Postpartum Time	n	Expression Frequency	Total Milk Production mL/24 h
Day 2	30	7 (5, 8)	22 (11, 65)
Day 4	37	7 (6, 7)	225 (80, 425)
Day 6	38	7 (6, 7)	532 (217, 685)
Day 8	31	7 (6, 7)	589 (335, 718)
Day 10	26	7 (6, 7)	600 (426, 724)
Day 13	26	6 (5, 7)	700 (400, 850)
Day 16	22	7 (6, 7)	710 (500, 1047)
Day 19	17	6 (5, 7)	770 (570, 1245)
Day 22	17	7 (6, 7)	725 (370, 1200)
Day 25	15	7 (6, 7)	758 (502.5, 957)
Day 28	11	7 (6, 7)	831 (608, 1129)
